# Universal design for learning in inclusive education policy in South Africa

**DOI:** 10.4102/ajod.v9i0.776

**Published:** 2020-12-15

**Authors:** Judith A. McKenzie, Elizabeth M. Dalton

**Affiliations:** 1 Department of Health and Rehabilitation Sciences, Faculty of Health Sciences, University of Cape Town, Cape Town, South Africa; 2 Department of Communicative Disorders, Faculty of Health Sciences, Dalton Education Services International, University of Rhode Island, Rhode Island, United States of America

**Keywords:** inclusive education, universal design for learning, South Africa, education policy, implementation

## Abstract

**Background:**

South Africa has undertaken the implementation of inclusive education as a vehicle for achieving enhanced educational outcomes and equity. Universal Design for Learning (UDL) is an instructional design framework that takes into account the wide range of variations in skills and abilities that exist across all learners, and provides a research-based set of principles and guidelines for inclusive curriculum development and delivery.

**Objectives:**

To locate UDL within the specific inclusive education policy context of South Africa and consider how this approach can support policy implementation. We have argued that UDL could serve as a strategy to link policy imperatives with classroom practice, enabling effective communication between the different actors.

**Method:**

We reviewed fundamental inclusive education policies in South Africa and research relating to their implementation, and how they configure support and curriculum differentiation. We then compared this understanding with that proposed by UDL and considered what could be gained in adopting a UDL framework.

**Results:**

We noted that UDL has several advantages in that it allows for a common language between education stakeholders and gives new meaning to the interpretation of levels of support.

**Conclusion:**

The implementation of inclusive education in South Africa could be enhanced by introducing the concepts of UDL into policy, research and teaching practice as a common language and vehicle for packaging support systems.

## Introduction

In 2011, the authors of this article jointly presented a workshop on the use of Universal Design for Learning (UDL) to a diverse audience of therapists, teachers and education managers and published the experiences. Given that this was a small reflective piece bringing together the conceptual underpinnings of South African education policy and the principles of UDL, as reflected in the workshop evaluations, we did not anticipate that it would be as widely read and cited as it has been. It became the most downloaded article from this journal by the second quarter of 2015 and, currently (June 2020), has 116 citations according to Google Scholar (accessed on 22 October 2020). In this article, we explore why the combination of UDL and inclusive education policy in South Africa has struck a chord with teachers and researchers, and we speculate as to how this synchrony can be further developed, with particular regard for how curriculum differentiation for different levels of support needs can be attained within the inclusive education system in South Africa.

We begin our discussion by examining the development of inclusive education policy in South Africa and then look at the critical role that curriculum differentiation plays within this policy and how the use of inclusive instructional design through the UDL framework can support this. We conclude with an argument for using UDL as a means to ensure appropriate flexible learning support, as opposed to fixed levels of support as proposed in South African policy.

## Background to inclusive education policy in South Africa

With the advent of democracy in South Africa, issues of curriculum change and provision of quality education to all children of all race groups assumed a high priority, largely because of the preferential treatment of white children under apartheid (Gwalla-Ogisi, Nkabinde & Rodriguez 2006). An overhaul of the entire education system was undertaken, and this included a reconsideration of special education and educational support. To this end, a consultative process occurred over several years which resulted in the development of Education White Paper 6: Special Needs Education: Building an Inclusive Education and Training System (EWP6) (Department of Education 2001), which outlines education policy for children with disabilities within a broad inclusive framework. This policy aimed to address the post-apartheid configuration of special education as one of racial inequity, limited educational access and segregation of children with disabilities. Furthermore, it was recognised that there are multiple causes of disadvantages. ‘Special needs’, it was argued, should therefore embrace not only issues of disability but also include issues of economic, social and linguistic contexts, and psycho-social challenges, such as the effects of human immunodeficiency virus (HIV). The term ‘barriers to learning’ was adopted by the National Commission for Special Needs in Education and Training (NCSNET) and the National Commission on Education Support Services (NCESS) (Department of Education 1997) to reflect the diverse nature of barriers and emphasise the removal of barriers through environmental or social interventions rather than through individualised therapy or treatment. In so doing, a systemic approach was espoused in which, according to Lomofsky and Lazarus (2001):

The factors which were conceptualised as barriers to learning and development were those which lead to the inability of the system to accommodate diversity, leading to learning breakdown or preventing learners from accessing educational provision. (p. 311)

An inclusive education system was adopted where all children can learn together within a seamless system of support that addresses not only disability but also a range of barriers to learning arising from poverty, inequality and other social conditions (Department of Education 2001). This system is built upon two pillars:

- A process for identifying barriers to learning and establishing support needs to address these barriers. This is presented in the National Strategy on Screening, Identification, Assessment and Support (SIAS) (Department of Basic Education 2014).
- Differentiation of the curriculum such that teachers can respond to diversity in their classroom and schools. Strategies to achieve this are presented in the *Guidelines to responding to learner diversity through curriculum and assessment policy statements in the Classroom* (Department of Basic Education 2011).

Education White Paper 6 moves decisively away from determining educational provision according to disability type and focusses rather on comprehensive support needs. In recognition of the fact that barriers to learning may arise at any level of the system, support needs are not only located within the learner but also at a systemic level in, for example, teacher education or curriculum differentiation (Department of Education 2001). Support is organised into different programmes, defined in the National Strategy as: ‘structured interventions delivered at schools and in classrooms within specific time frames’ (Department of Basic Education 2014:9). These programmes include the following:

- Provision of specialist services by specialised professional staff.
- Curriculum differentiation which includes adjustments and accommodations in assessment.
- Provision of specialised learning and teaching support material and assistive technology.
- Training and mentoring of teachers, managers and support staff.

For the purposes of this article, we will focus on the curriculum differentiation support programme, whilst recognising that our suggested approach will have implications for all the other programmes of support (especially training and mentoring of teachers). Within this understanding, three levels of support needs are identified, namely low-, medium- and higher-level support needs, with a progressive intensity, range and frequency of the different types of programme interventions. The three support levels are described within the programme of curriculum support as follows:

### Curriculum differentiation for different levels of support needs

Low support needs (LSNs) are those that can be addressed by short-term or one-off individual interventions and general capacity building of staff to meet a diverse range of learning needs. With regard to curriculum, there are adjustments made for LSNs to accommodate a range of functioning in the general education classroom to meet the learners’ varied needs. Adaptations are made at a classroom level and it is the role of a district-based support team to monitor the implementation and effectiveness of these adaptations on a regular, but infrequent, basis.

Moderate support needs (MSNs) are more specific and impactful and require longer-term interventions and consultative support. In terms of curriculum, additional planning time is needed from teachers to develop adapted instructional strategies and teaching support materials in consultation with curriculum advisors. These adaptations are monitored by the school- and district-based support teams. Because these adjustments may require additional resourcing, they would need to be processed at a district level.

High-level support needs (HLSNs) are intensive needs, requiring frequent, specific consultative support. This describes individual children’s needs that require a specialised environment and supports within the regular classroom, or a specialised classroom or a specialised school organisation, each with support materials, facilities and personnel that are available on a high-frequency and high-intensity basis. The curriculum support at this level consists of ‘complex and on-going adjustments to the regular curriculum programme’ (Department of Basic Education 2014:21).

Table 1 illustrates the different levels of curriculum adaptation skills according to the support needs that the teacher will be addressing.

**TABLE 1:** Teachers’ required knowledge of curriculum differentiation at different levels of support needs.

Low support needs	Medium support needs	High support needs
Teacher is skilled in curriculum differentiation (ordinary schools/general education classrooms).	Teacher requires additional training, planning time and consultation with experts (ordinary/general education classrooms and full-service schools).	Teacher requires specialised training and skills in learning variation, curriculum differentiation and specialised supports (full-service and special schools).

*Source:* Department of Basic Education, 2014, *National strategy on screening, identification, assessment and support*, Government Printer, Pretoria.

Whilst levels of support needs are associated with school placements (LSNs in ordinary schools; MSNs in ordinary and full-service schools; and HLSNs in full-service and special schools), the policy is very clear that rigorous efforts need to be made to address all levels of support in any type of school and to seek the necessary support provision in the ordinary school first. The SIAS strategy states that:

- ‘The learner has a right to be supported in his/her current school or the school closest to his/her home.
- Irrespective of the level of support required, every effort should be made to make the support available to the learner in his/her current/closest school.
- The District Based Support Team (DBST) may consider accessing Outreach Programmes from Full-Service Schools (FSS) and Special School Resource Centres (SSRC).
- The outplacement of the learner to an alternative setting to access a specialised support programme should be the last resort’ (Department of Basic Education 2014:61).

Whilst it appears to be logical that support provision is incremental, with each higher level of support incorporating the lower levels, this is unfortunately not made explicit in the policy. The possibility therefore arises that settings which offer high-level support are lacking in medium and low support provision. Therefore, it is clear that curriculum adaptation is complex and variable according to the level of support identified in the SIAS process. How then can the UDL approach assist with unpacking this complexity?

## Inclusive education policy and universal design for learning

Within South African disability policy, the White Paper on the Rights of Persons with Disabilities mandates a universal design approach defined as: ‘the design of products, environments, programmes and services to be usable by all persons to the greatest extent possible without the need for adaptation or specialised design’ (Department of Social Development 2016:15).

Applied to curriculum design, an approach that addresses the issues of importance for successful inclusion of students with differing support needs in education is UDL. The Global Education Monitoring report on inclusion and education promotes the UDL framework as being particularly relevant to a broad understanding of inclusive education as addressing barriers to learning, noting that: ‘The Universal Design for Learning concept encapsulates approaches to maximize accessibility and minimize barriers to learning’ (UNESCO 2020:120).

Universal design for learning was conceptualised in the early 1990s by the educators and researchers of the Center for Applied Special Technology, now known as CAST, in response to identified gaps between the needs of their students and their productive access to various instructional environments. Center for Applied Special Technology extended the previously existing principles of the conceptual framework of Universal Design (UD), through which physical environments could be designed for the widest range of differing access needs (Center for Universal Design 2008), and applied this way of thinking to educational environments. The UDL framework is based on neuroscientific research on how the brain functions (Rose & Meyer 2002). The three core principles of UDL, based on the recognition, strategic and affective neurological areas, address learner variation through proactive curriculum design. These principles specifically stated are: (1) multiple means of representation – presenting information and content in different ways; (2) multiple means of action and expression – differentiating the ways that students can express what they know; and (3) multiple means of engagement – stimulating interest and motivation for learning (CAST 2020; Meyer, Rose & Gordon 2014). Through the application of UDL principles and the accompanying UDL guidelines (CAST 2020), educators can conceptualise the many ways that instruction and materials can be varied to address the full spectrum of students’ differing learning needs – from low to high – and can design curricula and learning environments to address the needs of all students through a varied and comprehensive continuum of learning options and support choices.

Since its inception in the United States of America more than 25 years ago, UDL has grown to be widely recognised nationally and internationally as an important conceptual strategy and framework for the effective achievement of inclusive education (Davies, Schelly & Spooner 2013; Katz 2012; Meo 2008; Perez, Grant & Dalton 2016). Case study research reveals positive linkages between UDL implementation and inclusive education outcomes for high school students (Katz 2013), pre-K-12 and college students (De Freece Lawrence 2020) and online learning students (Bandalaria 2020). In the United States of America, the use of UDL to guide the development of inclusive educational supports and environments through multisensory learning centres has been shown to be effective in helping elementary students with learning, social and attention problems (Metcalf et al. 2009). Students with learning disabilities have made meaningful gains in reading comprehension and decoding skills, as well as gained access to the grade-level curriculum through the systematic use of the UDL framework (Cook & Rao 2018). Teachers and teacher candidates increased their abilities to effectively design and implement technology-infused lessons and incorporated more differentiated options and varied teacher strategies following training in UDL principles and guidelines. However, these studies also found that teachers need more experience in actually implementing the UDL principles in their classrooms (Courey et al. 2012; Harris & Yerta 2020). The most recent global education monitoring report entitled ‘Inclusion in education: All means All’ promotes UDL as an effective strategy for the inclusion of all children in education and notes that it has been adopted in education policy in Ghana and other low- to middle-income countries (UNESCO 2020).

There are also challenges to the implementation of inclusive education through the use of the UDL curriculum design framework and guiding principles that bear consideration. In a study carried out in South Africa, Song (2017) found that whilst teachers in low-resourced schools recognised the potential benefits of UDL, they expressed doubts about implementing the approach in their own schools. This highlights the need to adapt UDL to the particular context and the importance of teacher education. Bandelaria (2020) identifies the need for a holistic and comprehensive approach to UDL to overcome exclusion from learning opportunities and to contribute to a country’s social transformation and development. Arndt and Luo (2020) found that educators in China understood the need for providing varied means of learning for their students, but they felt that more knowledge and skills were needed to be able to fully accomplish this, or to integrate the UDL framework in their instructional practice. The real need for more professional development opportunities was identified. Research conducted by Reynor (2020) with pre-service teachers in Ireland revealed that whilst planning efforts for UDL integration did lead to more pupil-centred planning and better-informed views of the needs and capabilities of students with disabilities, participants noted that significantly more time was needed to prepare lessons that addressed the UDL framework and that they doubted they would realistically have time to do this throughout their lesson planning. Concerns also emerged regarding the use of technology which was ‘problematic at times, as Internet connectivity was not consistently available, especially in rural schools’ (p. 263).

Both benefits and challenges relating to UDL implementation in various settings, especially in still-developing and/or lower-income countries, should be seriously considered in any comprehensive inclusive education planning efforts.

## Linkages between universal design for learning and education policy

The conceptual framework of UDL has important linkages with the educational policy in South Africa which can assist in the planning and implementation of inclusive educational environments. We argue that this happens in several important ways and discuss these in some depth below:

- Universal design for learning provides a clear, understandable framework that facilitates communication between multiple team members. The UDL framework is interdisciplinary and clearly outlined in numerous texts (Grant & Perez 2018; eds. Gronseth & Dalton 2020; Meyer et al. 2014; Rose & Meyer 2002). Teachers, therapists and educational planners, educated in many approaches that strive to diversify curriculum and instruction such as multisensory instruction (Fernald 1943), taxonomy of learning (Bloom et al. 1956), multiple intelligences (Gardner 1983) and differentiated instruction (Tomlinson 1999) can leverage such knowledge and find a common language to talk about support for learners who experience barriers to learning. These principles are given expression and a framework for action in the core UDL principles of multiple means of engagement, representation and action and expression and the UDL guidelines that accompany them. In terms of South African policy, this can facilitate the development of individual support plans, as outlined in the policy on SIAS (Department of Basic Education 2014). The multi-disciplinary team, including the parents, can use the UDL framework to develop a common understanding of instructional supports that are needed for the child to succeed. The UDL framework offers options, means and examples that can help educators to implement desired and applicable learning approaches, such as those mentioned earlier. The three core principles of UDL guide educators to adapt their instruction in many different ways through the use of varied materials and approaches. Examples of these include the following: For multiple means of engagement, educators should provide options for recruiting interest, sustaining effort and persistenc and self-regulation; for multiple means of representation, provide options for perception, language and symbols and comprehension; and for multiple means of action and expression, provide options for physical action, expression and communication and executive functions. Additional details regarding options to be offered by UDL implementation are available in the UDL guideline grid (CAST 2018). Universal design for learning fosters professional collaboration and communication to achieve inclusive learning:
  ■ Whereas teachers speak the language of the curriculum, therapists are more steeped in medical or psychological terms. By paring down teaching and learning to the three processes of flexible methods of presentation, expression and engagement, all those working with the learner can collaborate with a common understanding (Dalton, Mackenzie & Kahonde 2012:6).
- The language of the UDL principles and guidelines is not specific to one setting or another, but rather flexible methods or ‘multiple means’ apply to all settings where learning can happen, whether therapeutic or educational. By reducing variations in:
  ■ terms, or ‘paring down’ through the shared use of the language of UDL, professionals of different disciplines can better understand each other’s needs and intentions regarding the implementing and sustaining inclusion. Universal design for learning is interdisciplinary in nature and refers not to one professional’s role and approach (for example, the role of the therapist as against that of the teacher) but rather to strategies for adaptation which can be used across disciplines.
  ■ In the South African context, Song (2017) found teachers were using some UDL practices but needed to develop their common language through professional development to realise the opportunity that UDL might offer in this context. In South Africa, large class sizes (up to 85 children per class in some rare cases) are likely to remain a reality for some time to come and teachers should therefore be trained in how to deal with this situation (Marais 2016). One strategy is to build diversity into learning and teaching at the planning stage, as specific adaptations for different learner needs are very taxing under these conditions. As a design framework, UDL starts from the planning stage and aims to design and deliver instruction for the widest range of diversity amongst learners by integrating variation in how teachers represent the content of the subject matter taught, how teachers engage students in learning through interest and motivation and how students show what they have learned in diverse ways and diverse products. The busy teacher can be prepared to deal with levels of diversity that are proposed within EWP6 through proactive instructional design.
- EWP6 places the teacher at the centre of the implementation of inclusive education and highlights the importance of ongoing professional development. In the UDL workshop mentioned above (Dalton et al. 2012), participants made a strong plea for further training in UDL. What became apparent is the attractiveness of one overarching framework for addressing a continuum of support needs through the curriculum, from low through to high support needs. Given the segregated special education system that continues to exist in South Africa today, because of a multiplicity of cultural and historical factors such as apartheid, family protectiveness, lack of awareness and/or lack of professional preparation opportunities, it becomes imperative for teachers to understand that disability support needs, although they might include specialised adaptations, should always incorporate lower levels of support, in terms of curriculum differentiation and planning for diversity and that these needs belong in the same conceptual framework of UDL. The system set up by the South African Council for Educators for mandatory professional development could include endorsement of well-designed and delivered short courses on UDL.
- Universal design for learning can be high-tech or low-tech, or even no tech. Whilst high-tech tools can offer many different options for varying content, means of response and learner engagement, these important areas can also be addressed through the variation of instructional strategies and use of simple tools and resources in creative ways by following the UDL guidelines and thoughtfully applying them in any given situation. This is reassuring for a South African population in which both more affluent and less affluent communities require quality education. With careful thought, planning and a full understanding of the UDL principles, both well-resourced and less well-resourced systems can cater to the diversity in their classrooms through the creative use of existing resources with a view to increasing equity and access.

## Understanding support needs through universal design for learning

As a result of the complexity of support needs, there is a tendency to view the levels of support as distinct from one another, rather than as a continuum of support. One of the unfortunate consequences of this view is that educators have come to view levels of support as associated with a certain school placement, despite repeated claims to the contrary within the SIAS policy. In a study conducted on teacher education needs, McKenzie, Kelly and Shanda (2018) found that many educators understood the support process as meaning that children with LSNs should attend regular schools, those with MSNs are best placed in full-service schools and those with high support needs in special schools.

In reporting on the implementation of inclusive education, the Department of Basic Education noted that ‘In contrast with the Special Schools, the highest incidence of learners with disabilities in ordinary schools are learners with Specific Learning Difficulties, Attention Deficit Disorder and Partial Sightedness’ (p. 19). This same report further notes the growth in special schools over the period of implementation of EWP6. These observations indicate that children with disabilities, who are viewed as having high support needs, remain excluded from the mainstream of education. This is a repeated finding in the South African context and raises questions of how disability is actually being addressed within inclusive education (Donohue & Bornman 2014).

Universal design for learning facilitates a continuum of support rather than discrete categories of support. The principles of UDL imply that variation in instructional design, delivery and support should be built into every classroom and lesson as planning for diversity is the starting point and not an add-on. Variation across students in their needs, capabilities, skills and interests is the norm, and not the exception (Meyer et al. 2014). Universal design for learning avoids any categorical descriptions and focusses teachers’ attention on learner variability and diversity from the start. Instead, a range of adaptations to meet learner needs and enable participation can be drawn upon. This avoids a situation where levels of support are associated with certain categories of adaptations but not others. Rather there is a recognition that, as stated in EWP6 (2001), *all* children need support to varying degrees at different times and *all* children need flexible support systems that will enable them to become better learners.

One such learning continuum model is outlined by Bray and McClaskey (2014) in their work on personalised learning, which describes the continuum to develop expert learners as moving initially from having student choice, to engagement, to motivation, to ownership, to purpose and finally to self-regulation. This continuum outlines a more ‘learner-centred’ environment, and the importance of such is described thus:

‘Learner-centred environments offer active and collaborative learning where learners are able to generate questions, organize inquiry projects and monitor their own products and progress’ (Bray & McClaskey 2014:168). Furthermore, such environments enable all children to benefit from adaptations when and where needed – adaptations are not only made for children identified as needing support but also for other children who can benefit from multiple means of representation, engagement and multiple means of action and expression. In a learner-centred environment, students become aware of and are encouraged and supported in exploring the varied options for accessing, integrating and expressing learning that has been built into the design of the learning environment. Such awareness develops each student as a ‘decision-maker’ on his or her own path to learning success and becoming an expert learner. The most recent version of the UDL guidelines, Version 2.2, emphasises the development of expert learners as the ultimate goal of education, defining expert learners as being purposeful and motivated, resourceful and knowledgeable, strategic and goal-directed (CAST 2018). These guidelines, when implemented with integrity, support a continuum of learning options in every classroom and work towards the outcome of making every student an expert learner.

## Conclusion

Universal design for learning can only be implemented through systemic change, and the possibility that it might be the driver of such change is an exciting one. However, this will require policy and planning support from educational administrators who will enable training and will recognise and support the best UDL practice.

We would therefore recommend the following strategies going forward:

- Teacher education programmes, in-service and pre-service, formal and informal, should include the principles and guidelines of UDL as a framework for developing classrooms that cater to the widest range of diversities.
- Support should not be thought of as low, medium or high and equated with placement options. It would be more useful to think of curriculum support in terms of what support each teacher needs to apply the principles of UDL to facilitate learning for every student and build a continuum of learning.
- Consideration should be given to the concept of ‘targeted universalism’ as an organising principle for the implementation of systemic change. As described by the Haas Institute (2019) at the University of California at Berkley:
  Targeted universalism means setting universal goals pursued by targeted processes to achieve those goals. Within a targeted universalism framework, universal goals are established for all groups concerned. The strategies developed to achieve those goals are targeted, based upon how different groups are situated within structures, culture, and across geographies to obtain the universal goal.

Such an approach can support the integration of UDL within the system of education, addressing the varied social, emotional and learning needs of differing groups whilst striving for the universal system-related goal.

Reasonable accommodation as defined in the White Paper on the Rights of Persons with Disabilities: ‘ensures that persons with disabilities enjoy, on an equal basis with others, all human rights and fundamental freedoms.… Reasonable accommodation support tends to be individual and impairment specific’ (Department of Social Development 2016:59). Support for inclusive education can be redefined in terms of UDL and reasonable accommodation. Although UDL can help us to plan for an increasingly wide range of diversities (as teachers receive training and support in these strategies), reasonable accommodation remains necessary for disability-related needs, such as sign language and/or Braille (United Nations 2006). Furthermore, this approach accords with disability policy in South Africa where EWP6 states that ‘Principles of universal design and reasonable accommodation provisioning must inform all new and existing legislation, standards, policies, strategies, plans and budgets’ (Department of Education 2001:107). In its General comment No. 4 (2016) on the right to inclusive education, the UN Committee on the Rights of Persons with Disabilities urges states to adopt a UDL approach to develop flexible and effective ways of adjusting to meet the requirements of every child, including those with disabilities. At the same time, the Committee recognises that if Article 24 of the convention, referring to education of people with disabilities, is to become a reality, then schools must also provide reasonable accommodation which meets the specific disability-related needs that learner might have. The provision of an accessible environment is necessary but may not be sufficient where specialised provision is required. Therefore, a continuum of supports ranging from generalised to specialised is recommended for educational systems to address the full range of learning challenges that exist. Whilst a wide range of learning needs can be met through flexible curriculum design, impairment-specific needs such as the use of Braille or learning South African Sign Language must also be catered for as reasonable accommodation within an inclusive education system.

Research on applications of UDL in the educational environments of countries such as South Africa and others around the globe would gather evidence of the effectiveness of various models for UDL implementation and should strengthen the argument of implementing UDL in low- and middle-income countries.

In this article, through an examination of support provision in inclusive education policy in South Africa, we have argued for a reconfiguration of the way in which we understand support as one of UDLs with reasonable accommodation for learners with disabilities.
